# Starting continuous improvement; creating a common understanding of stroke care delivery in a general hospital

**DOI:** 10.1186/s12913-024-11327-y

**Published:** 2024-08-06

**Authors:** Are Fjermeros, Geir Vegard Berg, Halvor Holtskog, Jos Benders

**Affiliations:** 1 Norway Department of Industrial Economics and Technology Management, Norwegian University of Science and Technology, Teknologiveien 22, Gjøvik, 2802 Norway; 2 Innlandet Hospital Trust, Lillehammer, Norway; 3 Norway Department of Industrial Economics and Technology Management, Norwegian University of Science and Technology, Alfred Getz vei 3, Gløshaugen, Trondheim 7491 Norway; 4 Centre for Sociological Research, KU Leuven, Parkstraat 45, Leuven, 3000 Belgium; 5 Inland Norway University of Applied Sciences, Elverum, Norway

**Keywords:** Cross functional, Disciplinary improvement, Enhanced coordination in hospitals, Quality improvement, Stroke care

## Abstract

**Background:**

Continuous improvement is based on fostering practitioners’ suggestions to modify their own work processes This improvement strategy is widely applied in healthcare but difficult to maintain. The cross-disciplinary nature of many care processes constitutes an extra impediment.

**Methods:**

The study had an explorative design with a qualitative single-case approach. The case presents a project to improve the treatment of patients with thrombotic stroke. Data was obtained via hands on involvement, documents, observations, and interviews with participants in a cross-functional improvement group. A thematic analysis method was employed.

**Results:**

Through learning how tasks were carried out in other disciplines, the participants developed a common understanding of why it took so long to provide treatment to stroke patients. These insights were used to implement practical changes, leading to immediate improvements in stroke care delivery. The results were fed back so that successes became visible. Participants’ understandings of the local context enabled them to convince peers of the rationale of changes, setting in motion a permanent improvement structure. The participants considered that mapping and then assessing the entire workflow across disciplines were relevant methods for improving the quality of patient care.

**Conclusion:**

Starting an improvement project in a cross disciplinary environment requires deep engagement on the part of professionals. A quintessential prerequisite is therefore the realization that the quality of care depends on cross-disciplinary cooperation. A facilitated learning arena needs to (1) create insights into each other’s colleagues’ tasks and process interdependencies, (2) increase understanding of how the distribution of tasks among specialist units affects the quality of care, and (3) frequently report and provide feedback on results to keep the process going.

**Supplementary Information:**

The online version contains supplementary material available at 10.1186/s12913-024-11327-y.

## Background

General hospitals tend to be organized in disciplinary silos. Such an organizational structure seriously impedes organizational learning and the ability to improve care processes. Improving care processes that involve multiple silos requires close coordination between those silos. This is hampered when individual silos are managed as stand-alone units, as tends to be the case.

This study explores and describes how a common understanding between disciplinary silos was created. By distilling key factors and mechanisms, we aim to further our collective knowledge on how to start continuous improvement projects in hospitals. To our knowledge, due to a dearth of papers on continuous improvement in healthcare and general hospitals in particular, this aspect has not yet been focused on. In their seminal paper *Why hospitals don’t learn from failure*, Tucker and Edmondson [1] came close in pointing to barriers between disciplinary silos, yet they did not relate this to continuous improvement. We show how healthcare professionals in different disciplines need to understand each other’s tasks as a starting point for improvement. This goes beyond cognition, as seeing how one’s tasks impact those of others in a cross-disciplinary treatment process also motivates involvement in improving care paths. Knowing the context of the entire workflow will create opportunities for reflection and improve practitioners’ assessments of the quality of the overall treatment process.

A general principle of the organizational structure of hospitals is to grouping practitioners into communities according to their medical competence. The surgery, neurology, internal medicine, radiology, etc. communities usually have their own manager, budget, and separate arenas for follow-up of outcomes. This organizational principle has led organizational researchers to classify hospitals as institutions with their own inherited ideological appeal and complex power relations constituted around various categories of expert knowledge [2]. Another typical feature of hospitals is that most outcomes are created through work processes that include contributions from many of the autonomous professional units, i.e. diagnosis and treatment of emergency medical conditions such as myocardial infarction, femoral neck fracture, stroke, and sepsis. In daily work, practitioners in the ambulance service, emergency departments, intensive care unit, radiology, medical laboratories, groups of doctors (MD), and wards will be involved in solving defined subtasks that are part of one continuous treatment path.

Research has revealed that the discipline-based organization of healthcare creates dysfunctions [3–7]. Studies report that lack of interaction and communication between the functional units leads to lower efficiency [8], discontinuity in the flow to the patients that decreases both productivity and satisfaction [9], duplication of work [7], and a lack of trust between employees working in different parts of the organization [6, 7].

A frequently cited study conducted at eight US hospitals highlights why it is difficult to find solutions to achieve better coordination across units [1]. It was found that practitioners experienced repeated problems due to a lack of coordination between professional units. However, these problems did not constitute a starting point for involving other units in the search for better solutions. Instead, practitioners solved problems as they arose by detouring or reworking inside their unit, without involving others. Results indicate that hospital-related cultural norms could explain the practitioners’ way of dealing with coordination problems. Firstly, efficiency concerns prompt staff to resolve issues quickly to prioritize patient care. Secondly, individual vigilance norms encouraged practitioners to solve problems without bothering other professional units. Finally, a widespread empowerment norm leads practitioners to solve problems themselves without involving managers.

These previous research findings have an impact on the conclusions in a summary review of organizational science in healthcare conducted by Mayo, Myers, and Sutcliffe [10]. Their systematic literature review of organizational science in the health sector describes research findings and trends through an analysis of the 730 most cited articles in the field over the past decade. They reported a lack of research on how different dependencies between subsystems create challenges and require solutions. These researchers argue that increasing specialization requires the development of new knowledge that can inform how different parts of the organization contribute to the overall care system [10].

The present study aims to answer the call from Mayo and colleagues. We argue that sustainable solutions need to create insights among practitioners so that they understand why coordination between professional units is relevant to the quality of patient services. Such knowledge creation will also need to develop an understanding of why accurate interaction between professional units is crucial for avoiding duplication of efforts and waste of valuable medical expertise. Based on this premise, we pose the following research question:

### What were the key factors and mechanisms in starting the continuous improvement of cross-disciplinary care processes in a general hospital?

We regard the initial period of an improvement intervention to be of specific interest for answering this question. Therefore, this study describes and analyzes changes in practitioners’ organizational problem understanding in facilitated improvement work aimed at improving stroke treatment in a Norwegian hospital. Several researchers describe such startup periods as a problem clarification phase [11–14]. Interviews with employees deeply involved in the start-up phase of this improvement effort provide information to increase understanding of how this happened in the present case.

## Design and methods

To gain an in-depth understanding of the underlying mechanisms behind the process of improving stroke treatment in the hospital we applied a single-case design. A case study is useful for understanding both complex social occurrences and organizations [17]. The case was selected because it contains comprehensive data relevant to the topic of the study. We explore and describe the startup period of cross-functional improvement work in a typical small Norwegian hospital. The improvement work was part of a larger effort to introduce lean thinking in the hospital. The lean approach aimed to develop the hospital’s ability to continually improve cross-disciplinary work processes by designing coherent flows with the least amount of waste [15, 16].

The hospital had chosen value stream analysis as the preferred methodology, and specialist units with practical tasks in value streams should be a part of improvement work. The narratives in this case are crucial aspects of the findings and constitute opportunities for obtaining complex knowledge and achieving a comprehensive understanding [15–17]. Utilizing multiple data sources is advisable in case studies because it allows diverse perspectives to be captured. The present case includes data from participant observations, individual interviews, documents, and statistics on the duration of treatment times. Participant selection is reported in the Case Study section.

### Data collection

We present data from two different time periods. The first dataset is a systematization of documents and observations from the period May–October 2013. This information was originally gathered to document work conducted in an inter-disciplinary group to improve stroke treatment. It includes meeting minutes, action lists, flowcharts, electronic mail exchanges, field notes, and statistics reporting outcomes. The field notes are important as observational data collected by the first author when working as an internal change agent in the cross-functional working group. The second data source includes interviews with five employees who participated in the group mentioned above. We were particularly interested in their reflections on how activities carried out in the start-up phase contributed to the development of organizational understanding. An interview guide was developed in collaboration between the first and second authors, please see Supplemental file 1. The interviews were conducted face to face by the first author between November 2022 and February 2023, lasted from 55 to 72 min, and were transcribed verbatim by the first author. The informants comprise two MDs, a radiographer, a nurse, and an employee of the ambulance service. Employees who participated in the cross-functional working group interviews voluntarily consented to participate in this study and completed an informed written consent form. SIKT (Norwegian Agency for Shared Services in Education and Research) has approved the research project in a decision by the Data Protection Officer at Innlandet Hospital *(Protocol nr.19,449,967).*

### Analysis

A thematic analysis [18] was conducted to identify the main themes and concepts related to the research question. Field notes and documents were chronologically organized to gain a timeline of activities conducted in the cross-disciplinary improvement group. The interview data was analyzed as a separate data set, which proved to be important. These post-working group reflections demonstrate sustained learning and support the themes identified through the observational documents. Secondly, they provided the basis for the development of two new themes (themes 3 and 4 in the result section). The data was assessed as a complete set and read several times in the search for patterns and themes. All authors were involved in the analysis work and discussed the findings at several meetings. The back-and-forth process between codes and themes involved reviewing relevant research and theoretical perspectives to help us understand the data. Development of the main and sub-themes was guided by the research question. Table 1 presents an example of how the analysis was conducted.

**Table 1:** Examples of how the thematic analysis was conducted (themes 2 and 3)

Codes	Categories	Themes
Together we found the problem areas Easier to help others when we understand their tasks and struggles Understood as we accessed the entire workflow Resistance in the professional departments Spent a lot of time convincing their peers Less resistance when understanding the “big picture”	Development of own organizational understanding Transferring new organizational insights	Organizational understanding developed over time through interaction with other practitioners Challenging to transfer understanding to colleagues

### Cross-disciplinary measure to improve stroke treatment

In May 2013, a concerned senior neurologist brought forward an issue to the hospital`s quality advisor. The MD had recently participated in a national stroke forum, which made her realize that patients suffering from stroke received poor-quality care at our hospital. According to the neurologist, poor organizing resulted in a delay in providing intravenous drug treatment to dissolve a blood clot in the brain. She called for immediate action, as patients with blood clots suffer extensive brain cell damage for every minute the condition remains untreated. The quality advisor agreed and suggested establishing an improvement group consisting of representatives from all specialized units involved in the treatment process. The neurologist objected, as she thought such a strategy would delay improvements. Instead, she sought the quality advisor’s help to reformulate procedures in the quality system, ensuring formal approval among hospital leaders, and dissemination of the updated procedures to all involved units with instructions to follow the new standard. After a discussion, the neurologist agreed to carry out the work using a group, on condition that the quality advisor took responsibility for gathering representatives from all the involved units.

At the next leader meeting, the quality advisor asked managers to support this work by selecting suitable representatives to form an interdisciplinary group. The selection should be based on two criteria. Firstly, the participants must possess good practical knowledge of the stroke treatment practice in the unit. Secondly, they must have a high professional reputation among employees in their unit.

The cross-functional stroke improvement group had its first meeting four weeks later. The participants consisted of nine skilled professionals without any formal leadership positions. They comprised a neurologist, a specialist nurse from the intensive care unit, a biomedical laboratory scientist, a radiologist, a radiographer, a nurse from the emergency department, a MD employed by the medical department, a local head of the ambulance service, and a quality advisor in the director’s leader group. Two of the participants in the interdisciplinary group were employed in cross-cutting divisions in the health trust. The representative from the ambulance service was affiliated with the Prehospital Services Division, and the biomedical laboratory scientist belonged to the Medical Services Division. In total, the group represented about 300 employees.

**Fig. 1:**
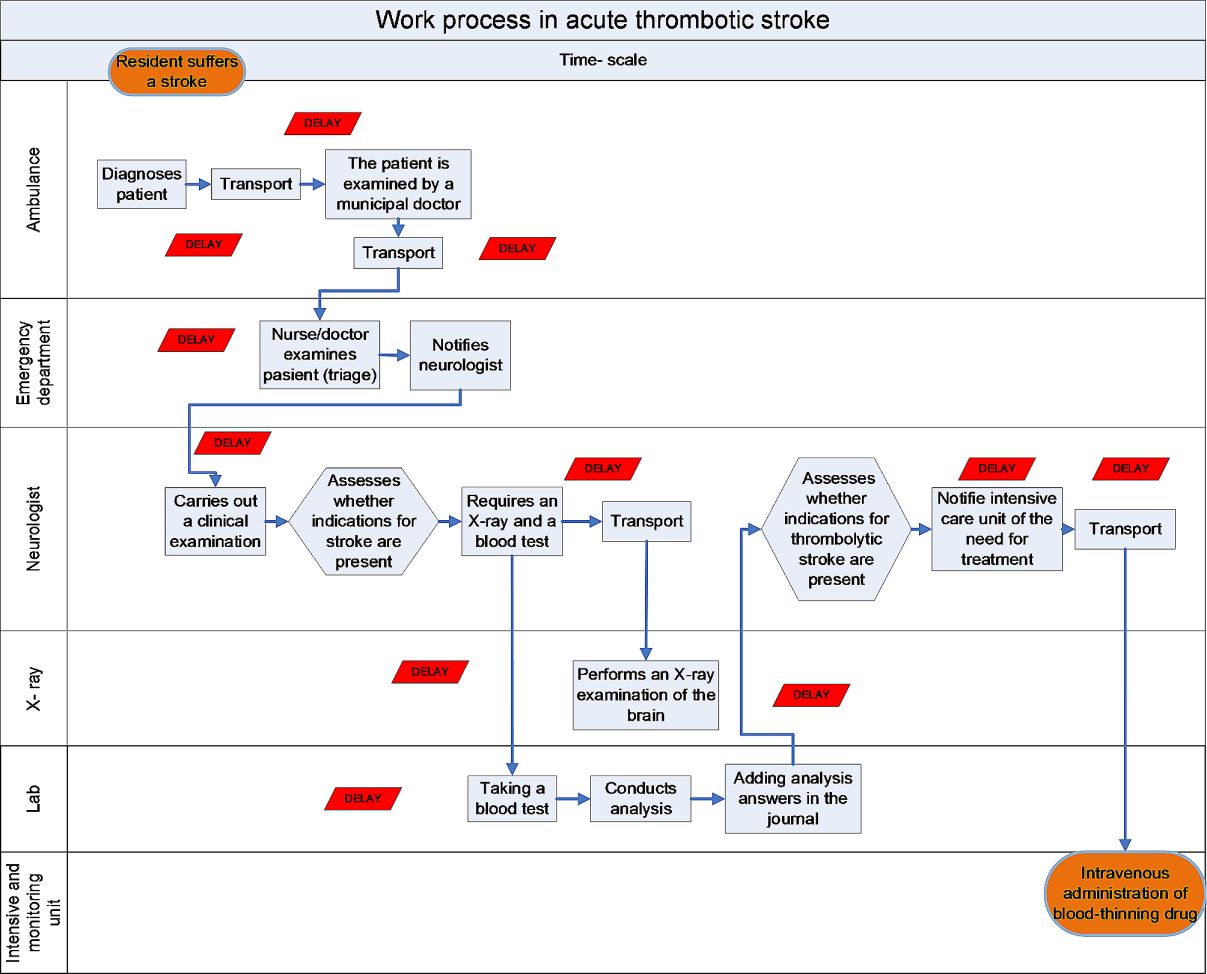
Simplified version of the employees’ mapping of tasks and associated bottlenecks in the treatment of stroke patients conducted in September 2013

Before the meeting, the quality advisor arranged the meeting room. Eight chairs were set up in a semicircle in the middle of the room. Yellow and red post-it sticky notes and a pen were placed in front of each chair. After all the participants had introduced themselves, the quality advisor put two notes on the wall. These represented the first (*ambulance picks up patient*) and last task (*patient receives treatment to dissolve the blood clot in the brain*) of the entire stroke work process at the hospital. Participants were told to write every work task their unit performed when treating stroke patients and place these notes on the wall. A couple of the professionals were alert and the quality advisor sensed that unrest was spreading in the group. One participant approached the quality advisor and said: *“Is the problem that you don’t you know how we work as professionals?”* One MD shook his head and said, *“We don’t have time for such nonsense. I believe we need to work more efficiently. Let’s start changing the stroke procedure*,* that’s why we’re here.”* The quality advisor ignored these inputs, as he deemed it essential to obtain detailed knowledge about actual work tasks. He had noticed that the ambulance driver and nurse from the emergency department were writing down tasks and asked them to put their notes on the wall and explain the tasks to the rest of the group. One note from the ambulance driver read: *“driving the patient to the municipal emergency room”.* One of the MDs now raised his voice and exclaimed: *‘No*,* you can’t do that*,* it delays the treatment’*. The representative from the ambulance service explained that they had been told by the municipal MDs to stop by the municipal emergency room as this would ensure that the patient was *“properly medically clarified”*. The quality advisor stated that this was important information and explained that the red notes placed in front of the chairs could be used to register what the participants perceived as bottlenecks in the care process. He told the MD who had provided input to write; *“delay*” on a red note and place it on the wall in connection with the ambulance driver’s description of the action. The next person to place task notes on the wall was the nurse from the emergency room. One note read “*conducts patient examination – takes vital parameters”.* Both the ambulance driver and the MD then told the nurse that this action was unnecessary and delayed treatment because these data had already been gathered. The emergency department nurse responded that this was a standard routine for all patients and nurses were instructed to do it by their manager. Again, the quality advisor requested that this should be written on a red post-it note and placed on the wall. Several of the clinical staff now began to write notes describing tasks performed in their unit and placed them on the wall. The neurologist hung up a note that read *“order emergency X-ray examination”*, and at the same time a red bottleneck note, which read; *“unnecessary waiting”.* The participant from the X-ray department now reacted, raising his voice in an irritated manner. He approached the neurologist and said: *“We`re doing the best we can*,* but equipment is always in use by another patient. Surely*,* we can’t just evict another patient who has nearly completed an extensive examination?”*. The quality advisor interrupted this somewhat heated discussion, stating that the issue could not be resolved at today’s meeting. At this stage of the meeting, activity in the group increased, participants brought up more yellow notes outlining tasks and several red notes describing bottlenecks. During the next two hours, more than 60 task notes were placed on the wall and more than 20 bottlenecks identified. Figure 1 presents a simplified version of the workflow mapping conducted by the group at this meeting.

At the end of this session, several participants commented that they had doubts about whether all tasks and bottlenecks had been included in the map. To verify the accuracy of the workflow, the group decided to collect more data by observing actual stroke practice before the next meeting.

Two weeks later, the cross-functional stroke improvement group met again. The participants had new information. A MD, involved in the treatment of a stroke patient, had noticed that treatment had been postponed due to the patient`s high blood pressure. The MD applied a red bottleneck tag on the wall that read “high blood pressure”. The ambulance driver replied, *«Perhaps ambulance personnel could prescribe drugs to patients to lower blood pressure before arrival at the hospital?”* The intensive care nurse had also experienced a new bottleneck, as treatment had been delayed because of the time required to insert a peripheral venous catheter. The ambulance driver replied that the ambulance crew *“had time to insert venous catheters during transport to the hospital”*, but he had another issue. Several of his colleagues thought it was difficult to assess stroke diagnosis because similar symptoms could occur in other medical conditions. The neurologist replied that the MD at the hospital could help in such cases. She said paramedics could “*get a direct number for the neurologist on duty at the hospital*,* and in case of doubt*,* they could call for professional support 24/7*”.

At the end of the second meeting, the participants agreed that the wall map now represented a credible picture of how tasks were distributed throughout the entire workflow. As the group assessed the entire care path, the quality advisor asked: “*Is this design well suited for providing the patient with the fastest viable treatment?”* The clinicians smiled exasperatedly and shook their heads, - all participants agreed that the design was weak. They summarized that delay in treatment was due to: patients being diagnosed several times by different actors, and four separate transport stages with delays between each stage. Finally, the process was slow because all units were unprepared when the patient arrived. Employees lacked knowledge of the arrival of a stroke patient and conflicts arose because everyone was busy attending other tasks. The quality advisor then posed the following question to the participants: *“Who decided that the hospital should organize stroke treatment in this way?”* Several of the employees laughed, and one said: *“It is obvious that whoever designed it was not a genius!”* At the end of the meeting, the group agreed to meet again two weeks later to work out a redesigned standard for stroke treatment.

## Results

The analysis revealed four main themes.

### Theme one

Practitioners acknowledged the relevance of utilizing a facilitated process to improve/enhance patient care. This theme includes the following subthemes: Use of an inter-disciplinary group, mapping the entire workflow identified the main problem as weak coordination between tasks, and assessing the entire workflow - ownership of new knowledge led to an obligation to contribute.

### Theme two

Practical changes with intermediate results.

### Theme three

Transferring new organizational insights to colleagues is challenging.

### Theme four

Development of an awareness of the importance of cross-disciplinary cooperation.

### Practitioners acknowledged the relevance of utilizing a facilitated process to improve patient care

#### Use of an inter-disciplinary group

The first meeting between the quality advisor and the experienced neurologist involved a dialogue that over time resulted in a common understanding of the change strategy. Firstly, they agreed that tardiness in stroke treatment was related to the weak coordination in task distribution among sub-units. Their understandings differed in terms of strategy. The neurologist wanted help from the quality advisor to distribute a revised stroke protocol initiated by a single unit to all departments for implementation. To maximize meaningful, sustainable change, the quality advisor introduced a strategy involving employees to develop their understanding. Thanks to the dialogue, the neurologist accepted that the quality advisor had expertise in how to organize work to achieve behavioral change among 300 hospital employees. Because the quality adviser received assistance from the hospital’s senior management team, the group was composed of practitioners who had relevant practical experience in the field to be improved, and high professional standing among employees in their unit. Many years later, the neurologist expressed her «new» understanding as follows: *“I had the answer*,* but not the recipe…. I’ve learned a lot from the work that we’ve done. It’s made me understand that there’s no point in getting someone to change if they don’t understand why*” (Quote from interview M.D November 2022).

The interdisciplinary group became an arena for the neurologist to explain the significance of changes for the future health of patients with thrombotic stroke. A participant expressed; “*I was surprised at the damage to patients if treatment was delayed by a few minutes.” “In this case*,* the benefits for the patient’s health were so obvious that it made it easier to make an effort in the work”* (Radiographer, December 2022).

#### Mapping the entire workflow identified the main problem as weak coordination between tasks

At the first meeting, participants brought different goals, motives, and understanding of problems to the improvement work, which were influenced by their own practice experiences. A common denominator for the focus on problems can be summed up by the statement *“At the beginning*,* everyone thought of themselves*” (radiographer, Dec.2022). One of the nurses recounted; *“The instruction from my unit was to not play an active part in this work. The reason was that stoke treatment could be a time waster in terms of us having to leave our unit*,* thus affecting the care of the other patients in the intensive care unit.»*. How can this focus be understood? At this stage of the improvement work, the stroke workflow was organized, so that units performed tasks in separate stages. This indicates that each unit only had experience of a limited part of the complete care delivery process.

Together, the practitioners used their clinical practice experience to create a wall image, revealing the entire stroke workflow. Later, they revised this description after collecting additional information from practice. Over time, it led members of the group to develop confidence that the image represented a truthful description of how work was carried out. The graphic wall picture made it possible for all participants to assess the entire work process at the same time. When they assessed the entire workflow, they found that units’ problems described as workflow bottlenecks (in Fig. 1) were part of another problem, namely a lack of logic and coordination in the distribution of tasks between units. Participants realized that different units attended to the same tasks at different times in the process (e.g., diagnosis and repeated documentation in medical records). Lack of communication (e.g., no notification to the next unit of the arrival of a stroke patient) created simultaneous conflicts and delays because equipment and personnel were busy. The map made it obvious to everyone that the delay in the treatment of patients was a consequence of the way the process was designed. A member of the improvement group remarked: *“To consider all stages of a workflow was new to us*,* and we discovered connections we hadn’t been aware of before.” (Ambulance driver*,* Dec. 2022)*. Several participants who were interviewed found it difficult to point out a particular time or event that changed their understanding of the problem. *“I learned a lot from this with mapping the bottlenecks*,* but we spent quite a lot of time formulating a description of reality that we all agreed was truthful. I think this (*authors remark: knowledge development*) takes time*,* and it’s important in itself.” (Nurse*,* Feb. 2023)*.

The mapping also helped participants identify cross-unit improvement opportunities. They even brought forward suggestions about how other disciplines could change how they performed their tasks to ensure speedy treatment. A paramedic participant commented: *“For us*,* it was very useful to gain an understanding of the entire work process*,* and not just our tasks. It created a better understanding of how we could work to contribute to better patient care.” (Ambulance driver Nov. 2022)*.

#### Assessing the entire workflow ownership of new knowledge led to an obligation to contribute

During the mapping process, the practitioners discovered that no one person or unit was responsible for the entire stroke process. They also understood that nobody deliberately created the poor design. They realized that the task distribution was something that had evolved, probably due to an accumulation of changes within each professional unit. At this point, these insights were not familiar to other professionals in the hospital. Together, these factors contributed to an understanding that if someone was to take responsibility for improving treatment for stroke patients, the group had to play an active role. A specialist nurse stated: “*I had a different feeling about whether we should be involved after the group had completed the workflow mapping. I went directly to my manager after this meeting and said that here we need to play an active part in the change work if stroke patients were to receive better treatment”* (Nurse- Feb. 2023).

### Practical changes with intermediate results

**Fig. 2:**
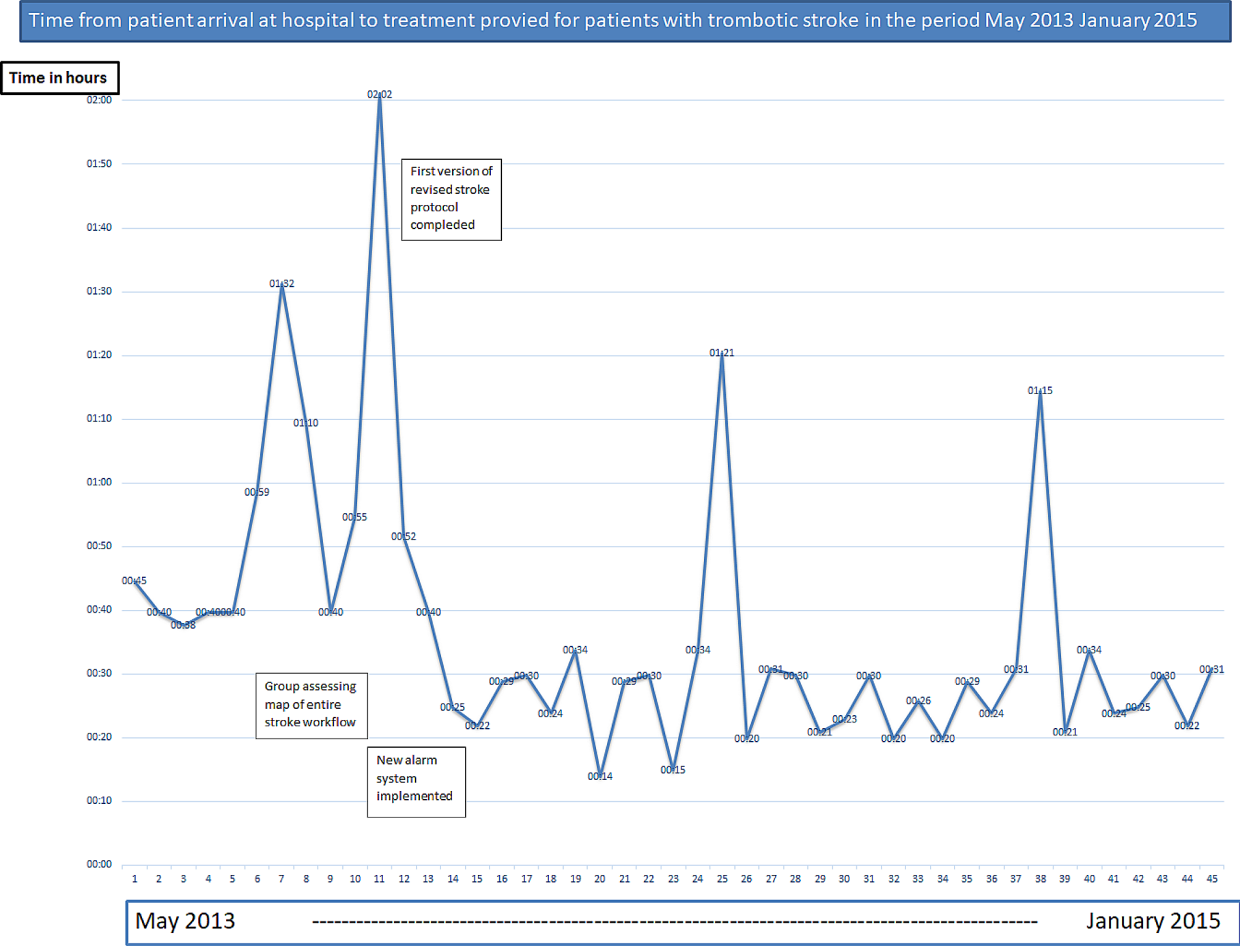
Door to needle times. *Source* Manuel registers made by the secretary of emergency medicine, based on the extracts from patient records

Practitioners’ new insights made them implement some practical changes in the task distribution that resulted in patients with stroke receiving much faster treatment to dissolve blood clots in the brain, as evident in “door to needle time” outcomes (Fig. 2). The rapid changes were possible because the improvement group was mandated by the director’s leader group and the quality adviser who led the work was part of the director’s management team. An agreement was made with all sub-unit managers that ongoing changes in the stroke procedures would be implemented without their formal approval. Instead, members of the improvement group discussed proposed changes with peers before implementing them in the revised stroke protocol. Important changes included ambulance personnel receiving assistance from a neurologist at the hospital to make a preliminary diagnosis. Subsequently, all units were notified before the patient arrived at the hospital, and patients were brought directly to the radiology department. There, professionals from various units gathered as a team to finalize the diagnosis and provide treatment.

Information from the reflexive interviews confirms that the changes implemented in 2013, were still maintained ten years later. Statistics from the National Stroke Registry *(Norwegian Stroke Registry | National Service Environment for Medical Quality Registries)* for the period 2016–2022 confirms that the results are permanent and place the hospital among the best at national level in the years 2021 and 2022.

### Transferring new organizational insights to colleagues is challenging

A common feature from the interviews is that the participants found it difficult to transfer their new organizational understanding to colleagues. *“It wasn’t just me who had to understand*,* others also had to understand”* (quotation from an interview with an MD in December, 2022). The participants’ new organizational understanding was developed through a process in which they first mapped out a complex work process consisting of more than 60 tasks. Because this was visualized on a wall, it became possible to assess whether the overall division of tasks was appropriate. This process was difficult to replicate in one’s professional unit. *“It was difficult to get others to understand the flowchart”* (interview nurse, January, 2023). One doctor stated: *“In the beginning*,* it was easier to discuss relevant changes with the professional staff in the working group because we had a common understanding of the flowchart”.* All participants said they spent quite a lot of time convincing colleagues of “the big picture”. They pointed to three factors that enabled them to succeed in developing employees’ understanding.

1. Focus on results; Door to needle times were published continuously – and motivated employees to find measures to reduce the time 2. Understanding local context. The participants considered their efforts necessary to develop understanding among peers in their professional unit. They had access to professional meetings, personnel meetings, or daily discussions with colleagues, and understood the operations of the professional unit. *“You or someone else at the hospital would not have been able to do this if you had brought a flowchart to our department. I understood how I had to do this in order to lower the level of resistance in our department”* (quotation from the interview with the radiographer, November 2022). 3. Finally, the practitioners thought it was of great importance that the knowledge had been developed in a group of peers. The interdisciplinary improvement group consisted of highly recognized clinicians, which gave participants the confidence to argue that new understandings was rooted in the clinic.

### Awareness of the importance of cross-disciplinary cooperation

The hospital has several work processes that cross professional hierarchies. It is not unreasonable to assume that weak coordination also creates problems in other cross-cutting work processes. The question is whether employees have developed a generalized understanding of the importance of coordination. If so, this would mean that they understand that weak coordination can be the cause of several quality problems at the hospital.

Several of the participants in the interdisciplinary improvement group suspected that other quality problems in the hospital could be understood by considering weak coordination in cross-cutting work processes. For example, one MD explained that *“a lack of quality in referrals from municipal MDs leads to the hospital staff spending a lot of time assessing low quality referrals*,* and this weakens the quality of patient services” (*MD, Nov. 2022). Similarly, an employee from the ambulance service believed that patients with a type of acute heart disease would receive improved health services if the entire patient pathway was better coordinated. Furthermore, they considered that the organizing principles used to improve stroke treatment would also be efficient for enhancing other cross-cutting work processes. However, the analysis of the data in this study does not provide sufficient support for claiming that the participants have developed a basic understanding that many quality problems in the hospital are due to poor coordination between units.

## Discussion

This study specifies key factors and mechanisms in the start-up period of cross-disciplinary improvement work in hospitals enabling all disciplines to pull in the same direction. The facilitated improvement working group process resulted in the identification of several tasks that emerged as opportunities for improving care across the stroke continuum. Reflective interviews confirmed the most important result of this improvement approach was a sustained insight/understanding on the part of practitioners that a poorly coordinated workflow resulted in a significant delay in the provision of effective treatment to stroke patients. To our knowledge, this has not been demonstrated in previous research. However, several studies have called for research that can provide such knowledge [10, 19–21] Previous studies also call for scientific approaches that can clarify how context influences the outcomes of organizational change [10, 16, 17, 19, 22]. This study presents an approach that includes a rich description of the context surrounding the start-up period of this improvement work. This provides opportunities for readers to assess whether the findings represent reasonable interpretations.

The findings can also be seen in light of theories of organizational learning. In a conceptual understanding of organizational learning, the mapping process can be described as an activity in which employees’ uncovered “theory in use“ [11, 12]. The VSM process revealed that none of the professionals knew the overall task solution. When given the possibility to access the entire workflow, it changed their understanding of what caused delays in the treatment of stroke patients. Could practitioners have achieved their new understanding without being directly involved in the improvement group? An alternative may have involved having process consultants map the entire workflow by walking the stroke pathway (following the patient’s footsteps). This could provide a workflow quite like the one produced in the interdisciplinary group (illustrated in Fig. 1), which could save time and allow representatives from the various units to reflect on the fully mapped workflow. However, the results of the present study indicate that this strategy is not a workable shortcut. Feedback from the participants in the improvement group reveals that the interaction with other professionals was crucial for the development of their understanding of the problem. These findings correspond with studies reporting that active involvement of and engaging the workforce seem to be driving forces behind maintaining continuous improvement in healthcare [23]. Several participants reported that their understanding changed over time because of the learning process. This can be understood by considering how their increased comprehension was obtained. Understanding was acquired when assessing the logical consistency in a complex graphic workflow consisting of more than sixty work tasks and more than twenty bottlenecks (see Fig. 1). It is difficult to recreate this reflection opportunity for employees who have not been part of the learning process. This finding is supported by a study reporting that healthcare professionals benefit most from learning styles that focus on the discovery of connections through own participation [24].

The interviews indicate that the employees’ new insights made them suspect that poorly coordinated cross-cutting processes resulted in other quality problems in the hospital. Such generalized organizational knowledge will be very valuable to hospitals that have numerous hidden cross-disciplinary processes in which the appropriateness of the distribution of tasks between units has not been assessed. However, the data analysis does not support the assumption that participants have developed a basic understanding that many other quality problems in the hospital are because of poorly coordinated cross-cutting processes. This is beyond the scope of the present study but might be an idea for future studies.

### Ethical and methodological reflections

Conducting research as an insider is challenging [25].The first author of this study had a defined responsibility for organizing the interdisciplinary working group and his participation has potentially affected the outcomes of this study (data collection, analyses, and results). The co-authors maintain positions as the first author’s supervisors. Throughout the development of the study, they challenged the first author’s interpretation of the data by proposing alternative interpretations. This led to reflections and discussions on what constitutes the most important findings of the study. As a result, the first author has been forced to reflect on why it is so important to involve practitioners. Or - why was it important that practitioners understood the necessity of changing the distribution of tasks between units?

Interview data was collected almost 10 years after the improvement work was initiated. It had the consequence that participants no longer remembered all the details from the start-up phase. Among other things, several participants mixed up the order of activities out at the time. This weakened the precision of the data but was somewhat remedied because the author had an excellent overview of the course of events based on meeting minutes and field notes.

## Conclusion

We set out to answer the following research question: *What were the key factors and mechanisms in starting the continuous improvement of cross-disciplinary care processes in a general hospital?*

Starting such an improvement project requires deep engagement on the part of healthcare professionals. A quintessential prerequisite is making healthcare professionals realize how the quality of care depends on cross-disciplinary cooperation. A facilitated learning arena needs to (1) create insights into each other’s colleagues’ tasks and process interdependencies, (2) increase understanding of how the distribution of tasks among specialist units affects the quality of care, and (3) frequently report and provide feedback on results to keep the process going.

### Electronic supplementary material

Below is the link to the electronic supplementary material.

Supplementary Material 1

## Data Availability

The datasets generated in the present study are not publicly available but are available from the corresponding author on reasonable request.

## Abbreviations

MD: Medical Doctor
VSM: Value Stream Mapping

## References

[1] Tucker AL, Edmondson AC. Why hospitals don’t learn from failures: Organizational and Psychological Dynamics that Inhibit System Change. Calif Manage Rev. 2003;45(2):55–72. 10.2307/41166165. 10.2307/41166165

[2] Doolin B. Enterprise Discourse, Professional Identity and the Organizational Control of Hospital Clinicians. Organ Stud. 2002;23(3):369–90. 10.1177/0170840602233003. 10.1177/0170840602233003

[3] Alves J, Meneses R. Silos mentality in healthcare services. In: 11th Annual Conference of the EuroMed Academy of Business. vol. 2018; 2018.

[4] Bento F, Tagliabue M, Lorenzo F. Organizational silos: a scoping review informed by a behavioral perspective on systems and networks. Societies. 2020;10(3):56. 10.3390/soc10030056

[5] Kreindler SA, Dowd DA, Dana Star N, Gottschalk T. Silos and social identity: the social identity approach as a framework for understanding and overcoming divisions in health care. Milbank Q. 2012;90(2):347–74. 22709391 10.1111/j.1468-0009.2012.00666.x PMC3460209

[6] Hajek AM. Breaking down clinical silos in healthcare. Front Health Serv Manage. 2013;29(4):45–50. 23858988 10.1097/01974520-201304000-00006

[7] McCartney M. Margaret McCartney: breaking down the silo walls. BMJ. 2016;354. 10.1136/bmj.i5199 27672074

[8] Vatanpour H, Khorramnia A, Forutan N. Silo effect a prominence factor to decrease efficiency of pharmaceutical industry. Iran J Pharm Research: IJPR. 2013;12(Suppl):207. 24250690 PMC3813367

[9] Drupsteen J, van der Vaart T, van Pieter D. Integrative practices in hospitals and their impact on patient flow. Int J Oper Prod Manage. 2013;33(7):912–33. 10.1108/IJOPM-12-2011-0487

[10] Mayo AT, Myers CG, Sutcliffe KM, Organizational Science and Health Care. Acad Manag Ann. 2021;15(2):537–76. 10.5465/annals.2019.0115. 10.5465/annals.2019.0115

[11] Argyris C. Double loop learning in organizations. Harv Bus Rev. 1977;55(5):115–25.

[12] Argyris C, Schön DA. Organizational Learning II: Theory. Method and Practice. 1996;2.

[13] Klev R, Levin M. Participative transformation: learning and development in practising change. Routledge; 2016.

[14] Lewin K. Field theory in social science: selected theoretical papers (Edited by Dorwin Cartwright.). Oxford, England: Harpers; 1951.

[15] Walshe K. Understanding what works–and why–in quality improvement: the need for theory-driven evaluation. Int J Qual Health Care. 2007;19(2):57–9. 10.1093/intqhc/mzm004. 17337518 10.1093/intqhc/mzm004

[16] Walshe K. Pseudoinnovation: the development and spread of healthcare quality improvement methodologies. Int J Qual Health Care. 2009;21(3):153–9. 10.1093/intqhc/mzp012. 19383716 10.1093/intqhc/mzp012

[17] Øvretveit J. Understanding the conditions for improvement: research to discover which context influences affect improvement success. BMJ Qual Saf. 2011;20(Suppl 1):i18–23. 10.1136/bmjqs.2010.045955. 21450764 10.1136/bmjqs.2010.045955 PMC3066695

[18] Braun V, Clarke V. Using thematic analysis in psychology. Qualitative Res Psychol. 2006;3(2):77–101. 10.1191/1478088706qp063oa. 10.1191/1478088706qp063oa

[19] Ramaswamy R, Reed J, Livesley N, Boguslavsky V, Garcia-Elorrio E, Sax S, et al. Unpacking the black box of improvement. Int J Qual Health Care. 2018;30(suppl1):15–9. 29462325 10.1093/intqhc/mzy009 PMC5909642

[20] Hill JE, Stephani A-M, Sapple P, Clegg AJ. The effectiveness of continuous quality improvement for developing professional practice and improving health care outcomes: a systematic review. Implement Sci. 2020;15(1):1–14. 32306984 10.1186/s13012-020-0975-2 PMC7168964

[21] Endalamaw A, Khatri RB, Mengistu TS, Erku D, Wolka E, Zewdie A, Assefa Y. A scoping review of continuous quality improvement in healthcare system: conceptualization, models and tools, barriers and facilitators, and impact. BMC Health Serv Res. 2024;24(1):487. 38641786 10.1186/s12913-024-10828-0 PMC11031995

[22] Moraros J, Lemstra M, Nwankwo C. Lean interventions in healthcare: do they actually work? A systematic literature review. Int J Qual Health Care. 2016;28(2):150–65. 26811118 10.1093/intqhc/mzv123 PMC4833201

[23] Kunnen Y, Roemeling O, Smailhodzic E. What are barriers and facilitators in sustaining lean management in healthcare? A qualitative literature review. BMC Health Serv Res. 2023;23(1):958. 37674182 10.1186/s12913-023-09978-4 PMC10483794

[24] Weggelaar-Jansen AM, van Wijngaarden J, Slaghuis S-S. Do quality improvement collaboratives’ educational components match the dominant learning style preferences of the participants? BMC Health Serv Res. 2015;15:1–13. 26087653 10.1186/s12913-015-0915-z PMC4473844

[25] Coghlan D, Casey M. Action research from the inside: issues and challenges in doing action research in your own hospital. J Adv Nurs. 2001;35(5):674–82. 10.1046/j.1365-2648.2001.01899.x. 11529969 10.1046/j.1365-2648.2001.01899.x

