# Supplementary material for: Starting continuous improvement; creating a common understanding of stroke care delivery in a general hospital
Source: BMC Health Serv Res. 2024 Aug 6;24:899. doi: 10.1186/s12913-024-11327-y (PMC11304807; doi:10.1186/s12913-024-11327-y)
Supplement: Supplementary file 1 — Supplementary Material 1 [file 12913_2024_11327_MOESM1_ESM.docx]

**Interview guide- conversations with participants in stroke improvement group, October 2022- February 2023**

**Partisipants formal Information:**

Name:

Department/unit:

Tasks:

How long have you been working at the hospital?

**What is learned?**

What experiences do you have from your work on improving the course of stroke patients?

What did you learn from this work (did you gain any new knowledge)?

Any follow-up questions:

- The workflow mapping work revealed bottlenecks or problems in the workflow, and changes were proposed that affected your specialist unit. This resulted in new procedures. How do you perceive that these changes were received in your own device?
- To what extent do you think the changes in task allocation were sensible changes?
- To what extent did the work give you insight/knowledge you did not have before?
- Prior to the workflow mapping, did you have knowledge of how other units solved their tasks?
- To what extent did this activity provide you with knowledge that would be useful in the further work of changing how the work should be organized?
- Any new/expanded knowledge was developed?
- What do you think were the most important changes that were made in terms of improving the services offered to this patient group?
- Has the improvement work given you any different understanding of the dependencies between different professional units in the hospital?
- Registrations carried out in the period 2013 – 2015 showed a reduction in the number of minutes from arrival at reception to effective treatment. What do you think were the most important changes that resulted in patients receiving faster health care?
- How are the results today?
- Why hadn't these changes in work flow been implemented earlier?
- Has the work of the group affected your own understanding of the dependencies between different functions in the hospital?
- Do you feel that the group had the legitimacy/authority to implement changes in work tasks for different academic units? Why?
- What creates a sense of security that the changes in task allocation (procedure) would gain acceptance in their own unit (that the changes were sensible)?
- How much time have you spent on the improvment work, outside of the meetings held in the improvement group?
- The knowledge you have acquired through this work (refer to what the interviewee has said), is this a knowledge that colleagues outside the working group have also acquired? If so, how have you contributed to this?

**Internal organisation of the improvement work**

- What experiences do you have in relation to how the improvement work was organized (refer to the specifics that the participant has been involved in)?
- What do you think is important to consider when organising improvement work that cuts across professional units in the hospital?
- Do you perceive that the improvement work was organised in a way that had an impact on what was achieved? If so, what was important?
- Could the changes have been implemented without the use of the improvement group?
- What activities have you carried out as part of your improvement work? (Overview from action lists can be used here to remind participants of tasks they have had in relation to planning and implementation of various measures, such as training of employees in their own unit, implementation of exercises, etc.)
- The group had regular meetings in the period autumn 2013 – autumn 2016. A review of the minutes shows that representatives from the professional units attended the meetings. Why was there so much loyalty in terms of attendance?
- Do you feel that the group had the legitimacy/authority to implement changes in work tasks for different academic units? Why?

*What significance do the following factors have the improvement work to yield results?*

- Composition of group (do you perceive that it was important that we gathered representatives from all disciplines when carrying out the improvement work)?
- Mandate?
- Group leadership?
- Use of methodology?
- Measurements of results?
- Anything else that matters?

**Learning to learn:**

Why was it a good idea to change the distribution of tasks between the units in stroke treatment?

Do you see any other bottlenecks/challenges in the hospital that can be linked to poor flow in collaborative processes?

Can experience from improvement work be used to improve the organisation of other patient pathways in the hospital? What will it take for us to achieve this?

Can the knowledge gained from the work to improve other cross-cutting work processes? In what cases might this be relevant – can you give some examples?

What does it take to make it happen?

Has the work on stroke improvement in any way changed the collaboration between your unit and other medical units at the hospital?
